# Clinical Reports of Diseases, Injuries, Surgical Operations, Etc., Etc. at the Buffalo Hospital of the Sisters of Charity

**Published:** 1857-07

**Authors:** Benj. H. Lemon

**Affiliations:** Formerly House Surgeon


ART. IV.— *Clinical Reports of Diseases, Injuries, Surgical Operations, &c., &c., in the Male and Female General Surgical and Private Wards of the Buffalo Hospital of the Sisters of Charity.* Care of Prof. HAMILTON, for the winter half year commencing Oct. 1st, 1856. By BENJ. H. LEMON, M. D., formerly House Surgeon.

Beginning with a general list I will include only those diseases, accidents, and operations of the more grave character, viz:

Anchylosis, various points; amaurosis; abscess, acute, in thoracic parietes, chronic of pelvis, and in various situations; burns from the clothing, three deaths from this cause; cataract, and operation, two cases; cystitis; caries of bones, operations for, including spine; contusions; caries of spine, with coxalgia; concussion of brain; cancer, including enchondroma of the maxillary bones of the left side, involving also the parotid gland; epithelial cancer of hand and lip; medullary cancer of the eye, also including the parotid, the fungous growth from the orbit being enormous.

Dislocations: two of the humerus downward into the axilla; one of the radius and ulna backward; epulis, or fibro-plastic growth on the superior maxilla, operation; ectropion, and operation; erysipelas phlegmonous; fractures; of the clavicle, single: of the humerus, single: radius and ulna, single: four; radius and ulna, single and comp., one; of the spinous processes of the ninth, tenth and eleventh dorsal vertebræ: neck of femur, single, three; shaft of femur, single, three; shaft of femur, double, compound, one; of the tibia and fibula, single, compound, one; of the tibia and fibula, single, two; frost bites; fistula-ani; gonorrhœa, simple, and complicated with stricture, with epididymitis, &c., &c.

Hydrocele, three cases, two operations; femoral hernia, strangulated and operation, recovery; necrosis of femur; paronychia.

Ophthalmia, including amaurosis, conjunctivitis acute and chronic; do sclerotitis, cataract, and two operations; iritis syphilitic; ulcer of cornea and hernia iridis, staphyloma, opaque cornea, albugo, nebula, leucoma; synovitis, three cases of knee joint; acute, spina bifida; synovitis, with hydrarthrosis; stricture of the membranous portion of the urethra, operation, successful result; strabismus, operations; syphilis in the primary, secondary, and tertiary stages of evolution; talipes, including first, second and third classes, namely, varus, valgus and equineus; synovitis and ulceration of the cartilages and caries of the articulating surfaces of the bones forming the knee joint: amputation at middle of the thigh, and fatal result; ulcers, chronic, from the effects of lightning: from varicose veins: syphilitic, syphilitico-phogedœnic scrophulous, &c.; varicocele, operations; varicose veins, operations for their relief; vesico-vaginal fistula, operation, and successful result; wounds: gun-shot, &c., &c., contused, lacerated, from various causes.

Died, effect of burns, three; necræmia from purulent absorption after amputation of thigh, one; croup, one; total, five.

CASE I.—*Ancient, Irreducible, Femoral Hernia, become Strangulated. Operation the ninth day after strangulation occurred. Successful result. Character, " Entero epiplocele."*

Mrs. Cath. Welch, æt. 50 years, Irish, a widow, much emaciated and in broken health.

Admitted Oct. 7th, 1856, to general surgical ward, female department.

Has an ancient femoral hernia of the right side, always partially reducible until nine days ago. In consequence of lifting too heavy a weight it suddenly became much enlarged, and resisted all her efforts to return it. A physician being called, applied himself to reduce it by the means generally adopted in such cases, but found himself unsuccessful. Symptoms of strangulation of the passive character ensuing, ordinary measures were adopted, that are supposed to favor the return of the gut. The protrusion continuing, however, the bowels not acting, the symptoms becoming more severe, and the patient growing exhausted, time wore away until the eighth day of incarceration, when stercoraceous vomiting supervened, and she was brought to the hospital.

On examination, a large oblong tumor was found occupying the right inguinal region, about six inches in its long, and three in its short diameter.

Having administered chloroform, a moderate quanty of which caused gradually a state of complete anæsthesia, without the usual struggling and delirium. Dr. Hamilton proceeded to operate: present and assisting, several members of the profession and the class from the Medical College.

The integuments were divided in the mesial line, in the direction of the long diameter of the tumor. The external epigastric artery bled little and required no ligation. The several successive layers of fasciæ were then divided by fine strokes of the scalpel, until the sac came into view, incision of which was followed by a copious discharge of yellow serum; both fasciæ and peritoneal sac were thickened. On laying open the sac a large amount of omentum presented itself, and posteriorly, and to the internal side of the omental mass, the intestine, of which the major portion of the tumor was composed, minute inspection now discovered extensive lymph exudations gluing the intestines together; also some very old adhesions; enlarged state of the veins distributed through the omentum; omentum itself of a pink color, and quite hard; and the strangulated knuckle of intestine of a dark claret color. The strictures, of which there were two, at the crural ring, caused by Gimbernaut's ligament and the falciform border of the pubic portion of the fascia lata, anterior layer, were pretty freely divided, cutting upward and a little outward.* The amount of protruding viscera being very considerable, a portion of the omentum, which was adherent to the sac, was cut away that the remaining contents might be more conveniently accommodated, and to lessen the danger of the inflammation continuing in the omental mass after its reduction.

Having ascertained that the strictures were relieved, the protruding intestine was gently returned within the cavity of the abdomen, going back with a gurgle, the omentum following. The lips of the wound brought together by three sutures, a pledget of lint, wet in cold water, was applied, and half a grain of morphia administered.

I saw her, ar the request of Dr. Hamilton, six hours after the operation; the bowels having moved naturally, had evacuated a very large quantity of liquid fæcal matter, apparently the same as that vomited previous to the operation.

Directed a change: a clean sheet to be drawn down from the head, as the soiled one was pulled toward the foot of the bed, thus preventing much disturbance and retaining the recumbent posture so necessary. Morph. half a grain this evening.

Oct. 8th. Slept little during the night. Pulse full and slow; tongue brown and dry; abdomen painful.

Repeat morphia; fomentations to abdomen.

9th. She looks brighter; slept well; pain in abdomen subsided.

Repeat morph.

10th. Dressed wound; improving.

Morph. ut ante.

11th. Comfortable; slept well; no stools since the seventh.

Ordered castor oil, half an ounce.

Dressed the wound, part of it having united by *primary adhesion.*

12th. Bowels have not responded.

Directed sal. Epsom, half an ounce.

---

* The reason for this is not assigned.

13th. Bowels relaxed last night.

Took out sutures and dressed the wound.

From this time she regained her appetite, and convalesced rapidly under tonics and a generous diet, requiring only tepid water enemata to regulate the bowels; by the first of Nov. wound had entirely cicatrised. Patient dismissed.

"In females hernia occurs most frequently from the twentieth to the fiftieth years: at fifty years the proportion is one in six.

"Average amount of mortality, nearly one-half the cases operated upon in the time of Sir Astley Cooper.

"Of seventy-seven cases operated upon by him, as reported by himself, thirty-six proved fatal; and of five hundred and forty-five cases recorded in the journals, and collected by Dr. Turner, two hundred and sixty are reported to have died.

"Later: Mr. Luke states that he has operated in eighty-four cases of hernia; in twenty-five of these the sac was opened, and eight died; in fifty-nine the sac remained unopened, and of these seven only proved fatal.

"Mr. Luke has shown that the ratio of mortality increases greatly in proportion to the length of time that the strangulation is allowed to continue. Of sixty-nine cases of strangulated hernia operated on within the first forty-eight hours of strangulation, twelve died; whilst of thirty cases operated on after more than forty eight hours had elapsed, fifteen died: in the former, 1 in 5.7: in the latter, 1 in 2.5.

"Mr. Erichsen also says, I have known a coil of intestine that had been but eight hours strangulated before the operation was performed, so tightly constricted as not to regain vitality after its reduction."—*Erichsen's System of Surgery.*

In the above case, therefore, the reasons for opening the sac were sufficiently urgent, namely, the long continuance of strictures, and the severity of the later symptoms generally.

Case II.—*Syphilis in the Secondary and Tertiary stages of evolution. Pseudo-membranous Croup and Death. Necropsy. Subject, a girl aged* 15 *years.*

This case presents anomalies exemplifying the extreme in each disease: the former incident generally to adult age, when not hereditary.

The latter (croup,) being obnoxious chiefly to those of ages ranging from ten months to five years, rarely occurring after the tenth year.

Mary Jane Burns, æt. 13 years. A short, stout, dark complexioned Irish girl, lymphatic temperament, from the Rochester Orphan Asylum.

Admitted to the Buffalo Hospital, Oct., 1854, with supposed scrophula, having a sore under her chin, and another at the inner angle of the right eye, causing fistula lachrymalis. The diagnosis was syphilis in the secondary stage, her previous history, (unsatisfactory,) youthful age, and some adverse opinions seemed to negative its correctness.

It was sustained, however, by the subsequent symptoms, as an account of the case, after the lapse of two years, will fully substantiate: the pathognomonie signs of syphilis in the secondary and tertiary stages of evolution being well marked.

*Present condition*, *Oct.*, 1856. Countenance pale, clay colored, waxy appearance; anorexia, secondary signs, a pustulo-crustaceous ulcer of a serpiginous character, under the chin, about one inch wide and three inches in length, with brown and yellow crusts on a dark claret-colored base; a sore of similar character at the inner angle of right eye and fistula-lachrymalis; enlargement of cervical glands; confluent herpetiform eruption on tha nates and thighs (inside.)

*Tertiary Signs* Nasal ostalgitis; nose flattened, obstruction of nares; cephalalgia; excoriated ulcer of throat and velum palati; aphonia; osteoscopic pains, chiefly of the anterior aspects of the tibiæ, and of the ulnar lateral aspects of the ulnæ. The parenchymatous inflammation causing nodular enlargemants of these bones, imparting to them a distorted and bent appearance.

She died suddenly in the night of the fourth of December; a patient in an adjoining bed, alamed by her struggles, called the Sister nurse, who found her moribund; dyspnœa had been observed the day previous, on being questioned, said she had taken cold.

*Necropsy* disclosed a yellowish-white pseudo-membranous exudation of firm consistence, lining the parietes of the larynx, trachea and bronchiæ; the examination was pursued no further.

Did the syphilitic cachexia predispose to the induction of croup?

Case III. — *Gonorrhœa, with Epididymitis supervening at the end of four months, without the cessation of the urethral discharge. Treated by diet, recumbent posture, cubeba, demulcent drinks. Cure in eight days, the Gonorrhœa disappearing previously to the resolution of the testis.*

Jacob Baker, æt. 21 yrs., German laborer; short stout, florid complexion.

Entered hospital, Dec. 2d, 1856, general ward; says he contracted his disease about four months ago; has had no treatment; kept about his work; discharge constant. Several days ago he felt pain in one of his testes, with sense of weight in perineum, and organ began to swell, became hard and very painful: this increased, until finding himself incapable of further exertion, he applied for relief. The whole testis is now involved in the inflammation; the epididymis, particularly the globus major portion and the spermatic cord being very painful; testis is about the size of a hen's egg. An abundant yellow puriform urethral discharge continues.

Directed to lie in bed; saturnine lotions, cubebs, one drachm, three times daily: increased the third day to two drachms, three times per diem; demulcent drinks; half diet.

The testicle enlarged no further, but on the contrary began rapidly to diminish in size, and by the eighth day of treatment had regained its normal condition, the cord remaining somewhat hard. The gonorrhœa had disappeared, however, previously to this. He remained in the house some days after this, without a recurrence of any of the symptoms. Dismissed.

"Ricord says that epididymitis, like the other symptoms of blennorrhagia, is neither a repercussion nor a metastasis. That the quicker the disease is arrested, the surer are we of immunity from consecutive accidents."

The article in *Cooper's Surgical Dictionary* on orchitis says, "This disease most frequently arises from previous gonorrhœa, and especially when the discharge has been injudiciously suppressed by astringent or saturnine injections into the urethra. When the tumefaction commences the pain and burning in urinating ceases, and the discharge retires altogether; but all these symptoms return so soon as the inflammation in the testicle is removed.

"Strangury, to an alarming extent, sometimes accompanies the swelling and stopping of the discharge, and hence many judicious surgeons invite the return of the secretion from the urethra, thus removing the hernia humoralis more speedily."

This case is at variance with these statements:

*First*, No injections had been administered.

*Secondly*, The orchitis supervened during the continuance of the discharge.

*Thirdly*, Pain and burning did not cease on tumefaction, nor did the discharge return after it ceased and the testitis had subsided.

*Fourthly*, It is fair to assume that the resolution of the inflamed testis was hastened by the cure of the urethritis and removal of irritation from this cause.

In support of this see following case to same effect.

CASE IV.—*Gonorrhœa. Testitis supervening at the end of three weeks. The urethral secretion continuing. Treated as in the preceding case, with the addition of injections of the sol. arg. nit. chrys. Cure of the Urethritis in ten days, and resolution of the Inflamed Testis at the end of the second week of the treatment.*

Mr. Simon S., æt. 30 years, unmarried; a strong man of sanguine temperament, employee of N. Y. C. R. R. Co., at Niagara Falls, admitted January 30th, 1857, to a private ward; says he contracted gonorrhœa about six weeks ago, and has been treated by a surgeon at the Falls, without benefit he thinks. Three weeks ago, the discharge being as copious as at any time, his right testicle grew inflamed; having taken medicine the secretion has ceased for the last week, until the day before his admission, when it recurred afresh, and with an aggravation of all his symptoms: burning pain on urinating, (ardor urinæ) desiring to do so more frequently; sense of weight and aching in perineum from before toward the anus; increased tenderness and swelling of testis.

His condition growing worse, determined him to apply to the hospital, in Buffalo, for relief.

The organ is now about the size of a large hen's egg; the epididymis and spermatic cord especially, hard and painful to the touch. The urethritis not being deemed sufficiently acute to contraindicate the use of injections, the sol. arg. nit. chrys., one grain dissolved in four ounces of water, was directed to be thrown up the canal four or five times daily, and retained for half a minute; cubeba, two drachms, three times daily; demulcent drinks; half diet; the recumbent posture, and the organ supported in a manner that its weight may not depend from the cord.

Under this treatment the urethral secretion disappeared in ten days, and by the end of the second week the inflamed parts had regained their normal state.

Dismissed February 17th. I heard from him some months subsequently, none of the symptoms had returned.

"Ricord says that the greater part of the symptoms to which blennorrhagia can give rise, never manifest themselves before the end of the first week, and it is from the second week, and most generally later, that we see these symptoms take place. Further, that the greatest number of these symptoms manifest themselves only in those cases where no treatment, or an insignificant one, has been made.

"Mr. Erichsen when writing on gonorrhœa, says of injections, much and unfounded prejudice exists against their use in the minds of many. That the bad consequences, such as stricture, inflamed testicle, &c., which have sometimes been referred to their use, have been rather due, either to the long continuance and to the severity of the disease itself, than to the remidies employed, or to their application at too early a stage, or of too great strength. It is in long standing cases of gonorrhœa in which the discharge continues for months, or even years, that stricture results, not in cases of ordinary duration, and in these it is the result of chronic inflammatory thickening of the mucous membrane, and has no more to do with the injections than the copaiba or salines the patient may have taken.

"Gonorrhœal inflammation of the testis invariably affects only one testis, and commences in the epididymis, whence it extends to the body of the organ.

"It usually occurs in those individuals who have a lax and long pendulous scrotum. It seldom sets in before the third week after the occurrence of the gonorrhœa, but may occur at any period during the continuance of the discharge, though it is more frequent between the fifth and sixth weeks, than at any other time. In many instances it is referred to some slight injury — a blow or squeeze received during the continuance of the discharge; but in other cases it would appear to arise from extension of inflammation along the ejaculatory ducts, and in others again to a kind of metastasis of the morbid action from the urethra and the testis. The fact of the disease commencing in the epididymis may be advanced in support of the first opinion, whilst the fact that the discharge usually ceases when the inflammation of the testicle comes on and returns as it subsides, may be adduced in support of the doctrine of its metastatic origin. — *Erichsen's Surgery.*

(To be Continued.)

